# Staged biventricular repair in a premature neonate with critical aortic stenosis, severe mitral regurgitation, and fetal hydrops: a case report

**DOI:** 10.1186/s44215-024-00148-4

**Published:** 2024-04-29

**Authors:** Yuta Teguri, Takashi Kido, Koji Miwa, Tomomitsu Kanaya, Shigemitsu Iwai, Hisaaki Aoki, Sanae Tsumura

**Affiliations:** 1 Department of Cardiovascular Surgery, Osaka Women’s and Children’s Hospital, Izumi, Osaka Japan; 2 Department of Pediatric Cardiovascular Surgery, National Cerebral and Cardiovascular Center, Suita, Osaka Japan; 3 Department of Pediatric Cardiology, Osaka Women’s and Children’s Hospital, Izumi, Osaka Japan

**Keywords:** Critical aortic stenosis, Left atrial dilatation, Fetal hydrops, Bilateral pulmonary artery banding

## Abstract

**Background:**

The surgical management of critical aortic stenosis, mitral regurgitation, and left ventricular dysfunction is a significant clinical challenge. Whether left ventricular function will recover to support systemic circulation after the relief of aortic stenosis is a concern. In this setting, surgical or balloon aortic valvotomy combined with bilateral pulmonary artery banding and atrial septectomy may allow time for left ventricular adaptation, while the systemic circulation is supported by the right ventricle through the ductus arteriosus. We describe the case of a premature neonate with critical aortic stenosis, severe mitral regurgitation, and fetal hydrops who successfully underwent staged biventricular repair after bilateral pulmonary artery banding, atrial septectomy, balloon aortic valvuloplasty, and stent implantation for ductus arteriosus.

**Case presentation:**

A 29-year-old female was referred to our hospital at 25 weeks of gestation with fetal echocardiography findings of critical aortic stenosis, severely impaired left ventricular function, severe mitral regurgitation, and restrictive foramen ovale. At 33 weeks of gestational age, the baby was born via cesarean delivery. Prostaglandin E1 infusion was immediately initiated, and the neonate underwent emergecy bilateral pulmonary artery banding and atrial septectomy. On the second day, a balloon aortic valvuloplasty was performed. The neonate underwent stent implantation to open the ductus arteriosus and multiple-balloon aortic valvuloplasty. At 4 months of age, he underwent biventricular repair consisting of surgical aortic valvuloplasty, atrial septal defect closure, bilateral pulmonary artery debanding, and ductus arteriosus ligation. At 1 year of age, he underwent the Ross –Konno procedure. Six years after the operation, the patient’s general condition was stable, and the patient is doing well.

**Conclusions:**

Staged biventricular repair was successfully achieved in a premature neonate with fetal hydrops and critical aortic stenosis associated with severe mitral valve regurgitation and left ventricular dysfunction.

## Background

Patients with critical aortic stenosis (AS) typically undergo balloon aortic valvuloplasty (BAV) or surgical aortic valvuloplasty, if biventricular repair is feasible. Both procedures have been reported to have acceptable outcomes; however, the presence of impaired left ventricular (LV) function and severe mitral regurgitation may increase the risk of mortality and morbidity.

Fetal hydrops, defined as abnormal fluid accumulation in at least two different fetal cavities, is a rare complication, with a reported prevalence of 1 in 1700–3000 pregnancies [1]. The presence of cardiovascular abnormalities is a major cause of nonimmune fetal hydrops. In particular, structural lesions that result in right atrial pressure and/or volume overload are the most common etiologies of fetal hydrops with a poor prognosis [2].

Herein, we describe the case of a premature neonate with critical AS, impaired LV function, severe mitral regurgitation, and fetal hydrops treated with a staged biventricular repair. The first stage involved palliation of atrial septal defect (ASD) enlargement, bilateral pulmonary artery (PA) banding, stent implantation in the ductus arteriosus, and BAV, followed by the definitive repair by surgical aortic valvuloplasty, ASD closure, bilateral PA debanding, and ligation of the ductus arteriosus.

## Case presentation

**Fig. 1:**
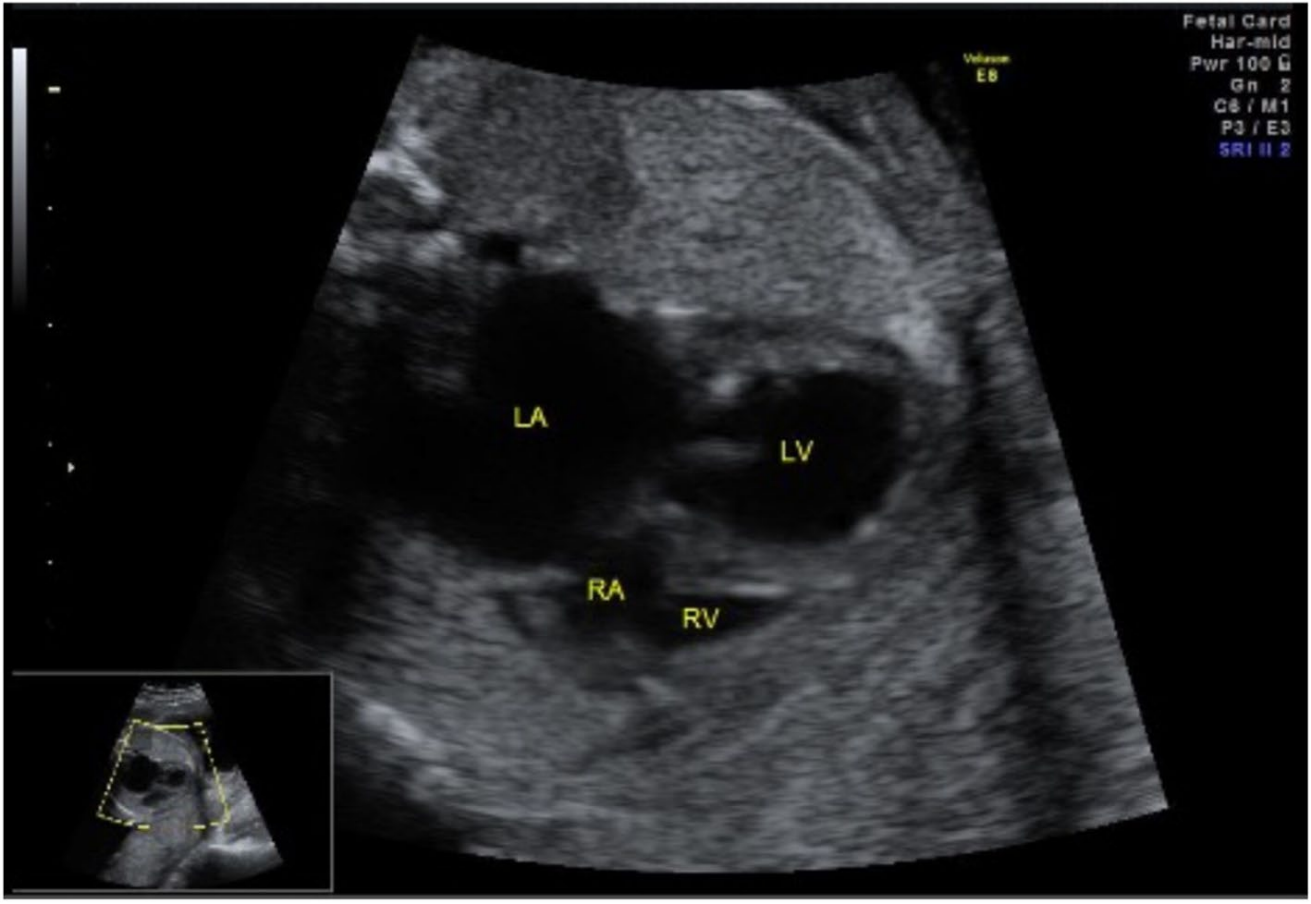
Fetal echocardiogram images at 25 weeks of gestation. Fetal echocardiogram in a four-chamber view demonstrating left atrial (LA) enlargement with bowing of the atrial septum into the right atrium (RA). The right ventricle (RV) is compressed by the dilated left ventricle (LV)

**Fig. 2:**
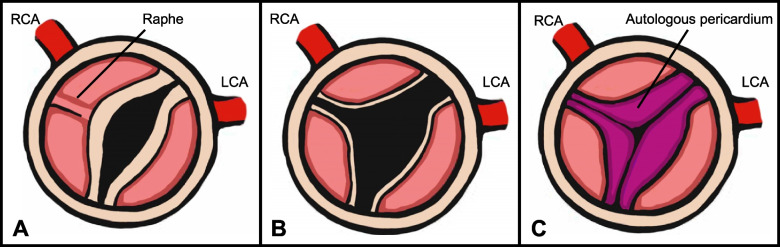
Aortic valvuloplasty illustration. **A** Severely thickened bicuspid aortic valve. **B** Raphe resection and slicing of the aortic cusps. **C** Cusp extension using autologous pericardium fixed with glutaraldehyde

**Fig. 3:**
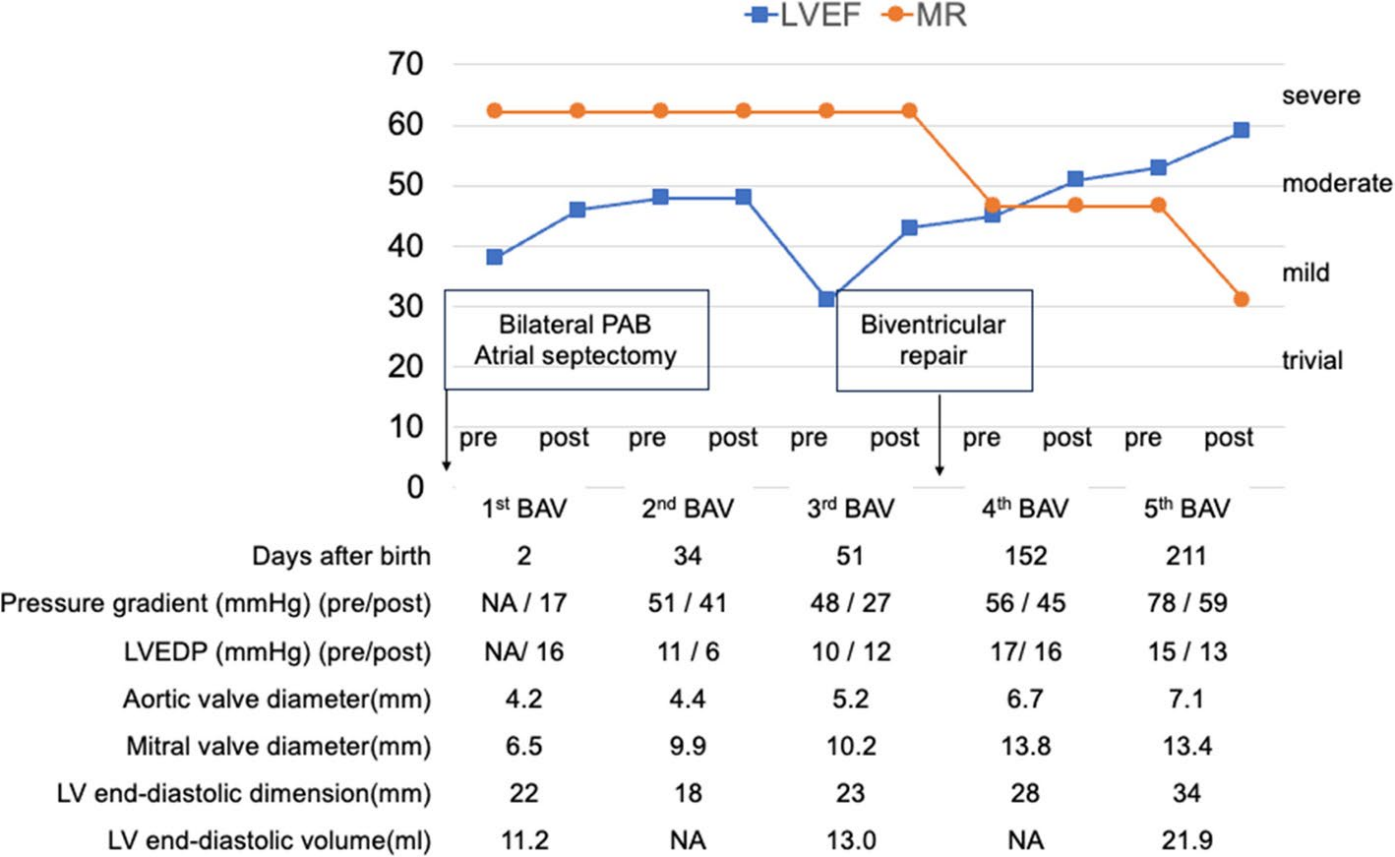
The time course change of echocardiographic data before and after BAV. BAV, balloon aortic valvuloplasty; LVEF, left ventricular ejection function; MR, mitral regurgitation; PAB, pulmonary artery banding; LVEDP, left ventricular end-diastolic pressure; LV, left ventricular

A 29-year-old female was referred to our hospital at 25-week gestation. Fetal echocardiography showed critical AS, severely impaired LV function with an ejection fraction of 35%, severe mitral regurgitation, and a restrictive foramen ovale. The left atrium (LA) was severely dilated, and the right heart structures were compressed (Fig. 1). The fetus was diagnosed with hydrops based on pleural effusion and subcutaneous edema. At 33 weeks of gestation, the patient was born via cesarean delivery, with a body weight of 2650 g. The Apgar score was 2/7. The patient presented with general edema and severe cyanosis (arterial oxygen saturation, 70% with 100% oxygen inhalation). Echocardiography showed restrictive ASD, severely dilated LA, and a reduced LV ejection fraction (LVEF) of 24%. The diameter of the aortic valve annulus was measured as 3.5 mm (Z score: −4.41) on echocardiography. Prostaglandin E1 infusion was immediately initiated, and emergent bilateral PA banding was performed. Concomitantly, the ASD was enlarged to a diameter of 8 mm using cardiopulmonary bypass. On the 2nd day after birth, BAV was performed thorough internal jugular artery using an IKAZUCHI balloon (4 mm × 2 cm) (Kaneka Medix Corporation). After BAV, the pressure gradient through the aortic valve was 17 mmHg, and the LVEF increased from 38 to 46% without the development of aortic insufficiency. On the 34th day, second BAV was performed using TREK balloon (4.5 mm × 1.5 cm). The next day, stent implantation was performed for the stenosis of the ductus arteriosus. On the 51st day, echocardiography revealed a decreased LVEF of 31%. A third BAV was performed using a TREK balloon (5 mm × 1.5 cm) (Abbott Medical), which resulted in an increase in LVEF to 43%. At the age of 4 months and body weight of 3.59 kg, surgical aortic valvuloplasty, ASD closure, bilateral PA debanding, and ligation of the ductus arteriosus were performed. The aortic valve is severely thickened and bicuspid without any injuries. Aortic valvuloplasty was performed with raphe resection, slicing and partial resection of the aortic cusps, and cusp reconstruction using an autologous pericardium fixed with glutaraldehyde (Fig. 2). Due to severely impaired LV function, the patient was managed with extracorporeal membrane oxygenation for 9 days postoperatively. On the 16th postoperative days, echocardiography revealed residual AS with deteriorated LV function. Cardiac catheterization using a sterile balloon (7 mm) (Boston Scientific Corporation) showed residual AS with a gradient of 56 mmHg, which decreased to 45 mmHg after BAV. Two months after surgery, the patient developed a hepatoblastoma and underwent hepatic resection and subsequent chemotherapy. Before hepatic resection, cardiac catheterization showed residual AS with a gradient of 78 mmHg, and a fifth BAV was performed. The time-course changes in LVEF, degree of mitral regurgitation, pressure gradient through the aortic valve, and left ventricular end-diastolic pressure, aortic annular size, mitral annular size, and LV size are shown in Fig. 3. After complete remission of the hepatoblastoma, the Ross–Konno operation was performed at the age of 1 year and body weight of 4.56 kg using a 14-mm polytetrafluoroethylene conduit with a bulging sinus for the right ventricular outflow tract. Intraoperatively, the repaired aortic valve was severely thickened and had decreased mobility. Six years after the operation, the patient is doing well and in a stable general condition. His cardiac function is good with LVEF of 51% with mild-moderate mitral regurgitation on echocardiography. Recent cardiac catheterization showed no pressure gradient through the LV outflow tract and an acceptable left ventricular end-diastolic pressure of 9 mmHg.

## Discussion and conclusion

Fetal AS with significant mitral regurgitation can cause severe LA enlargement, leading to right ventricular compression and hydrops formation. The prognosis of this complex is very poor, with a reported survival rate of 10% [2]. A dilated LA causes obstruction of the pulmonary venous return and compression of the right heart structure. Therefore, immediate postnatal LA decompression is necessary to rescue these patients. While catheter balloon atrial septectomy and BAV are commonly performed, we decided to perform emergency surgical atrial septectomy using cardiopulmonary bypass in the present case because of the patient’s unstable general condition with severe cyanosis. Postoperatively, multiple BAV improved the LV function and eventually achieved biventricular repair. Concomitant surgical aortic valvuloplasty was not performed, because it would require prolonged cardiac arrest, which may worsen the patient’s condition. Furthermore, the aortic valve was so small that surgical aortic valvuloplasty would not be significantly more effective than BAV. There have been considerable debates whether neonatal AS should be treated primary aortic valve surgery or BAV. While aortic valve surgeries are frequently needed after BAV when performed in the neonatal period, survival after neonatal BAV was reported to be acceptable [3].

In neonates with critical AS and severe LV dysfunction, bilateral PA banding, atrial septectomy, and prostaglandin E1 infusion (or stent implantation for ductus arteriosus) provide temporary right ventricle-dependent systemic circulation while decompressing the LV. Hammel et al. reported on four neonates with critical AS and severe LV dysfunction who underwent staged biventricular rehabilitation consisting of surgical aortic valvuloplasty, bilateral PA banding, and atrial septectomy, followed by patch closure of the ASD, ligation of the ductus arteriosus, and removal of PA bands [4]. After stage 1, LV function recovered in three patients, and they underwent stage 2, resulting in a good outcome with biventricular circulation in two patients. Unrestrictive atrial communication and a widely open ductus arteriosus may lead to significant LV unloading [4]. The interval between stages 1 and 2 in their series was 14–33 days, and no patients showed clinically relevant LV regression over this short period. A similar approach has been applied to critical AS in neonates with borderline LV or very low birth weight. Brown et al. reported a hybrid approach of bilateral PA banding, atrial septostomy, and ductal stenting. This approach is performed 4 weeks after BAV as a bridge to biventricular repair in neonates with critical AS and borderline LV [5]. Kimura et al. reported that the ascending aortic approach for BAV and concomitant bilateral PA banding was feasible in a very low birth weight neonate with critical AS and poor LV function [6].

In neonates with critical AS and LV dysfunction, whether LV function recovers enough to support systemic circulation after BAV is a concern. In this setting, surgical or balloon aortic valvuloplasty, concomitant with bilateral PA banding and atrial septectomy, may provide adequate time for LV adaptation and serve as a bridge for biventricular repair. In particular, neonates with critical AS and severe mitral valve regurgitation may benefit from this staged procedure because of the severely dilated LA, the presence of retrograde pulmonary hypertension, and compressed right ventricular structure. Neonates require time to stabilize their general condition through decompression of LV while maintaining adequate systemic circulation. Although we completely closed ASD at the time of biventricular repair, one could argue that fenestration for atrial septum can decrease LV volume load and prevent progression of LV dysfunction. This point should be determined based on LV function as well as mitral valve annular size.

The following two concerns should be considered when applying this approach. First, this staged procedure may interfere with LV growth due to atrial septectomy, resulting in LV unloading. After stage 1 surgery, LV function should be carefully monitored, and a Norwood-type operation should be considered if LV function appears inadequate for biventricular repair. Second, LV diastolic dysfunction is a late emerging complication of BAV for critical AS, even without significant residual LV pressure or volume overload. Robinson et al. reported on four teenagers who presented with significant LV diastolic heart failure after successful palliation of AS in early infancy [7]. While the LV end-diastolic pressure decreased to 9 mmHg at the most recent follow-up in our case, further close surveillance of LV diastolic function is warranted.

## Data Availability

The datasets used and/or analyzed in the current study are available from the corresponding author upon reasonable request.

## Abbreviations

AS: Aortic stenosis
BAV: Balloon aortic valvuloplasty
LV: Left ventricular
ASD: Atrial septal defect
PA: Pulmonary artery
LA: Left atrium
LVEF: Left ventricular ejection fraction
